# Time Trends in Excess Mortality Among Individuals With Bipolar Disorder in Finland, 2000–2023: A Nationwide Register Study

**DOI:** 10.1111/bdi.70147

**Published:** 2026-07-05

**Authors:** Julia Rimmington, Sonja Lumme, Kimmo Suokas, Mai Gutvilig, Marko Elovainio, Christian Hakulinen

**Affiliations:** ^1^ Department of Psychology, Faculty of Medicine University of Helsinki Helsinki Finland; ^2^ Finnish Institute for Health and Welfare Helsinki Finland

**Keywords:** bipolar disorder, mortality, time trend

## Abstract

**Objectives:**

To investigate excess all‐cause mortality among individuals with bipolar disorder in Finland and examine time trends in the mortality gap between individuals with bipolar disorder and the general population during 2000–2023.

**Methods:**

Individuals diagnosed with bipolar disorder during 1975–2023 in Finland were identified using the Care Register for Health Care and linked to population register data on mortality and demographics. Mortality was examined using age‐standardized rates per 100,000 person‐years. Relative excess mortality was estimated using mortality rate ratios (RRs) comparing individuals with bipolar disorder with the general population, stratified by sex and age group.

**Results:**

During 2000–2023, individuals with bipolar disorder had substantially higher all‐cause mortality than the general population. The age‐standardized mortality rate ratios were 2.85 (95% CI 2.77–2.92) for men and 3.25 (95% CI 3.14–3.36) for women. Mortality declined over time in both groups, and relative excess mortality decreased, although improvements were not uniform across age and sex groups. Relative excess mortality was particularly elevated among younger individuals and was more pronounced in women than in men.

**Conclusions:**

Individuals with bipolar disorder in Finland experience substantial excess all‐cause mortality compared with the general population. Although relative excess mortality declined during 2000–2023, it remains considerable. Because causes of death were not examined, these all‐cause estimates do not identify specific drivers of excess mortality, especially in younger age groups where relative excess mortality is highest.

## 1 Introduction

Bipolar spectrum disorders are episodic mood disorders with a lifetime prevalence of 1% to 2% [1]. They are defined by recurring and alternating episodes of mania or hypomania and depression, with periods of remission in between [2]. Individuals with bipolar disorder are more likely to have lower socioeconomic position [3, 4, 5], exhibit neuropsychiatric [6] and social impairments [7], and have a high burden of medical comorbidities, all of which are associated with an increased risk of severe health problems, including mortality [8, 9, 10].

Since the 1960s, advancements in several essential domains, such as healthcare and medicine, education, income, nutrition, and sanitation, have extended the life expectancy of the global population by twenty years [11, 12]. However, individuals with bipolar disorder may not have benefited to the same extent. Compared to the general population, individuals with bipolar disorder have a life expectancy reduced by 9 to 20 years [8, 13, 14, 15, 16]. While recent studies on varying populations have typically highlighted natural causes as the primary factor contributing to reduced life expectancy in individuals with bipolar disorder [10, 17], it appears that during the past decades, external causes of death have been the primary contributor to reduced life expectancy for persons with bipolar disorder in Finland [18]. Notably, while natural causes of death are more prevalent overall, external causes, such as suicide, accidents, and other unnatural causes, are disproportionately common among younger individuals with bipolar disorder. As deaths at younger ages reduce life expectancy more than those occurring later in life, the high proportion of external causes of death among younger individuals with bipolar disorder substantially contributes to their reduced life expectancy. In Finland, for instance, external causes account for 73% of deaths among individuals with bipolar disorder aged 15–44, and 38% among those aged 45–64, highlighting their substantial contribution to premature mortality in this population [18].

Research has investigated excess mortality among individuals with bipolar disorder across various study settings, consistently identifying elevated all‐cause and cause‐specific mortality [10, 19, 20, 21, 22, 23, 24]. However, a critical aspect that warrants further exploration is how the elevated mortality rates among individuals with bipolar disorder have evolved over time. Population‐based studies on time trends in excess mortality among individuals with bipolar disorder are scarce, have mainly used inpatient data, and report varied results: Studies utilizing Danish secondary care register data reported a widening mortality gap between persons with bipolar disorder and the general population from 1995 to 2014 [22, 23]. Likewise, a UK‐based cohort study, which employed primary care data, identified an increase in excess all‐cause mortality among individuals with bipolar disorder compared to the general population from 2006 to 2014 [19]. In contrast, a population‐based study employing Swedish inpatient data from 1987 to 2010 observed declining all‐cause and cause‐specific mortality for individuals with bipolar disorder over the study period. However, these improvements were more pronounced in the general population, leading to a widened mortality gap [20]. Lastly, a study using Finnish, Swedish, and Danish register data from 1987 to 2006 found that the life expectancy gap among individuals admitted to hospitals for any mental disorder narrowed over time, except for Swedish men with mental disorders [25]. This study, however, encompassed various mental disorders, making it challenging to determine whether similar trends apply specifically to bipolar disorder.

To address gaps in prior research, we employ Finnish register data, encompassing both inpatient and specialist outpatient care. We aim to determine whether and to what extent individuals with bipolar disorder in Finland experience excess all‐cause mortality compared with the general population and whether the mortality gap changed over time from 2000 to 2023. The results of this study may help inform public health and clinical interventions aimed at reducing excess mortality among individuals with bipolar disorder.

## 2 Methods

### 2.1 Data

The study period was January 1, 2000, to December 31, 2023. The cohort comprised individuals diagnosed with a primary diagnosis of bipolar disorder (ICD‐10: F30–F31; ICD‐9: 2962, 2963, 2964, 2967A; ICD‐8: 2961, 2963) in Finnish inpatient and specialist outpatient healthcare services between January 1, 1975, and December 31, 2023. Specialist outpatient data were available from 1998 onward.

The study population comprised non‐institutionalized individuals aged 10–74 years residing in Finland at the beginning of the study period. Individuals diagnosed with bipolar disorder between January 1, 1975, and December 31, 1999, were included as prevalent cases at baseline. After baseline, individuals entered follow‐up on January 1 of the calendar year in which they turned 10 years of age or from the beginning of the calendar year of immigration to Finland. Individuals were followed until December 31 of the year preceding their 75th birthday, emigration, death, or the end of the study period. The comparison group comprised all non‐institutionalized Finnish residents aged 10–74 years at some point during 2000–2023 who were not yet classified as having bipolar disorder.

Individual‐level linkages were performed between healthcare registers and Statistics Finland population registers containing mortality and demographic data. Mortality data included dates of death for all deaths occurring during follow‐up. The Institutional Review Board of The Finnish Institute for Health and Welfare evaluated the project's research plan in the light of ethical practices (THL/1477/6.02.01/2016§751), and Statistics Finland (TK‐53‐1696‐16) and the Finnish Institute for Health and Welfare permitted the data linkages. Informed consent is not required for register‐based studies in Finland.

### 2.2 Statistical Methods

Age‐standardized mortality rates per 100,000 person‐years were calculated and are presented with 95% confidence intervals. Rates were estimated separately by sex and age group (10–24, 25–64, and 65–74 years). Age strata were selected to ensure adequate statistical power among individuals with bipolar disorder while enabling meaningful comparisons across groups. The chosen categories reflect clinically and socially meaningful life stages—adolescence and early adulthood, working age, and older adulthood—which may differ in health profiles and mortality risk. As a sensitivity analysis, mortality rates and rate ratios were additionally calculated using 5‐year age bands across ages 20–64 years to assess heterogeneity within the broader age categories.

To reduce random annual variation and ensure sufficient numbers of events within strata, data were compiled into six 4‐year periods (2000–03, 2004–07, 2008–11, 2012–15, 2016–19, and 2020–23). Rate ratios (RRs) comparing mortality between individuals with bipolar disorder and the general population were calculated to estimate relative excess mortality, with 95% confidence intervals.

To evaluate temporal trends in mortality and relative excess mortality, Poisson regression models were fitted to death counts with person‐years included as an offset. The calendar period (4‐year intervals) was modeled as a continuous variable. Models included bipolar disorder status and an interaction between bipolar disorder status and calendar period. Analyses were conducted separately for men and women and stratified by age group.

All analyses were conducted using Stata/SE 18.0 (StataCorp LLC, College Station, TX, USA) and R version 4.4.1 (R Foundation for Statistical Computing, Vienna, Austria).

## 3 Results

During 2000–2023, individuals with bipolar disorder contributed 310,985 person‐years among men and 410,457 person‐years among women, compared with 51.9 million and 51.4 million person‐years in the general population, respectively (Table 1). A total of 5255 deaths occurred among men and 3380 among women with bipolar disorder, corresponding to 1.8% of all deaths among men and 2.4% among women during the study period. The proportion of the population diagnosed with bipolar disorder increased from 0.21% to 0.95% among men and from 0.23% to 1.35% among women between 2000 and 2023.

**TABLE 1:** Cohort characteristics, 2000–2023.

	Age group	Bipolar disorder population		General population	
		Men	Women	Men	Women
Person‐years	10–74	310,985	410,457	51,903,170	51,395,665
	10–24	18,037 (6)	36,626 (9)	11,818,671 (23)	11,264,757 (22)
	25–64	258,774 (83)	328,690 (80)	34,475,357 (66)	33,708,009 (66)
	65–74	34,174 (11)	45,142 (11)	5,609,142 (11)	6,422,899 (12)
Observed deaths, n	10–74	5255	3380	281,566	140,366
	10–24	97 (2)	95 (3)	6494 (2)	2496 (2)
	25–64	3616 (69)	2110 (62)	150,377 (53)	67,781 (48)
	65–74	1542 (29)	1175 (35)	124,695 (44)	70,089 (50)

*Note:* Person‐years and observed deaths are shown by age group and sex for individuals with bipolar disorder and the general population in Finland, 2000–2023. For age‐specific rows (10–24, 25–64, and 65–74 years), values in parentheses represent percentages within each population and sex group aged 10–74 years.

**FIGURE 1:**
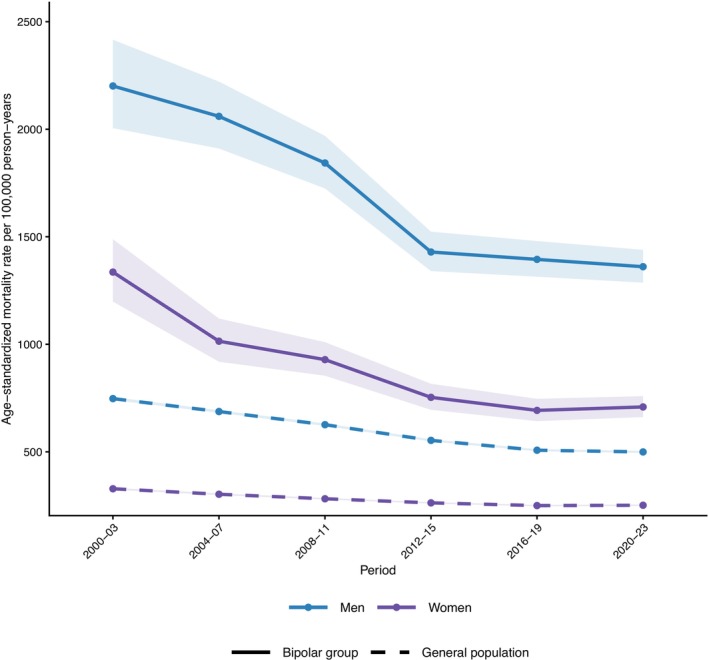
Age‐standardized all‐cause mortality rates by sex and period, 2000–2023. Rates are expressed per 100,000 person‐years with 95% confidence intervals among individuals with bipolar disorder and the general population aged 10–74 years in Finland. Numerical values underlying this figure are provided in Table S1.

Age‐standardized all‐cause mortality rates are shown in Figure 1 and Table S1. In the general population, mortality among men declined from 747 per 100,000 person‐years (95% CI 741–754) in 2000–2003 to 499 (95% CI 495–504) in 2020–2023. Among women in the general population, rates decreased from 328 (95% CI 324–332) to 252 (95% CI 248–255). Among men with bipolar disorder, mortality declined from 2201 per 100,000 person‐years (95% CI 2005–2416) to 1361 (95% CI 1287–1439). Among women with bipolar disorder, rates declined from 1336 (95% CI 1199–1488) to 709 (95% CI 662–759).

In formal trend analyses, mortality declined significantly over time in the general population, decreasing by 3.7% per 4‐year period among men and by 1.0% among women (both *p* < 0.001). Mortality among individuals with bipolar disorder also declined, but at a steeper rate, by 7.7% per 4‐year period among men and by 11.9% among women (both *p* < 0.001). Consequently, relative excess mortality among individuals with bipolar disorder declined by 4.3% per 4‐year period among men and by 11.0% among women (*p* < 0.001 for both).

Despite these declines, individuals with bipolar disorder experienced consistently higher mortality than the general population throughout the study period (Table 2). For 2000–2023 combined, the age‐standardized mortality rate ratio was 2.85 (95% CI 2.77–2.92) among men and 3.25 (95% CI 3.14–3.36) among women. Period‐specific estimates remained elevated across all intervals.

**TABLE 2:** Age–standardized rate ratios of all–cause mortality by sex and period, 2000–2023.

Period	Men	Women
2000–2003	2.95 (2.68–3.23)	4.07 (3.65–4.54)
2004–2007	3.00 (2.78–3.23)	3.35 (3.03–3.70)
2008–2011	2.94 (2.75–3.15)	3.30 (3.03–3.59)
2012–2015	2.58 (2.42–2.76)	2.87 (2.65–3.11)
2016–2019	2.75 (2.59–2.92)	2.77 (2.57–2.99)
2020–2023	2.72 (2.57–2.88)	2.82 (2.63–3.02)
2000–2023	2.85 (2.77–2.92)	3.25 (3.14–3.36)

*Note:* Cells show age‐standardized rate ratios (RRs) with 95% confidence intervals comparing individuals with bipolar disorder with the general population in Finland.

**FIGURE 2:**
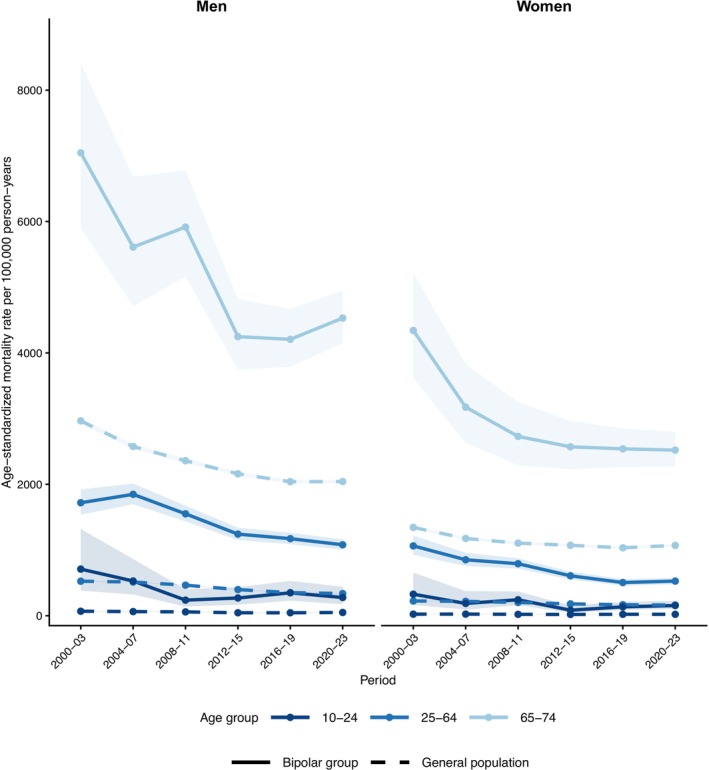
Age‐standardized all‐cause mortality rates by age group and sex, 2000–2023. Rates are expressed per 100,000 person‐years with 95% confidence intervals among individuals with bipolar disorder and the general population in Finland. Age groups are 10–24, 25–64, and 65–74 years. Numerical values underlying this figure are provided in Table S2.

Age‐specific mortality patterns are shown in Figure 2 and Table S2. Mortality declined over time in the general population across all ages and in most age groups among individuals with bipolar disorder.

In Poisson regression models, mortality in the general population declined significantly across all age groups in both sexes (all *p* < 0.05). Among individuals with bipolar disorder, mortality also declined significantly in most age groups. Among men, mortality decreased by 16.7% per 4‐year period in those aged 10–24 years (*p* = 0.005), by 10.9% in those aged 25–64 years (*p* < 0.001), and by 8.0% in those aged 65–74 years (*p* < 0.001). Among women, mortality declined by 16.1% per 4‐year period in the 25–64‐year group (*p* < 0.001) and by 7.9% in the 65–74‐year group (*p* < 0.001), whereas the decline among women aged 10–24 years was not statistically significant.

Relative excess mortality among individuals with bipolar disorder decreased over time in those aged 25–64 years in both sexes and in women aged 65–74 years. Among men, the decline was 2.8% per 4‐year period in the 25–64‐year group (*p* = 0.006), with no significant change in those aged 10–24 or 65–74 years. Among women, relative excess mortality declined by 11.2% per 4‐year period in the 25–64‐year group (*p* < 0.001) and by 3.9% in the 65–74‐year group (*p* = 0.034), whereas no significant change was observed among women aged 10–24 years.

Age‐specific rate ratios comparing individuals with bipolar disorder to the general population remained elevated across all age groups throughout the study period (Table 3). For 2000–2023 combined, the highest relative excess mortality was observed among individuals aged 10–24 years, with rate ratios of 5.42 (95% CI 4.90–5.99) among men and 6.85 (95% CI 6.15–7.62) among women.

**TABLE 3:** Age‐standardized rate ratios of all‐cause mortality by age group, sex, and period, 2000–2023.

	Period	Age group		
		10–24	25–64	65–74
Men	2000–2003	5.07 (3.92–6.56)	2.37 (2.29–2.46)	1.45 (1.34–1.56)
	2004–2007	6.05 (4.81–7.61)	2.44 (2.36–2.52)	1.57 (1.47–1.67)
	2008–2011	4.64 (3.72–5.78)	2.57 (2.49–2.66)	1.69 (1.59–1.80)
	2012–2015	5.58 (4.49–6.93)	2.61 (2.52–2.70)	1.73 (1.63–1.85)
	2016–2019	5.52 (4.27–7.14)	2.70 (2.59–2.82)	1.83 (1.71–1.97)
	2020–2023	9.06 (6.27–13.09)	3.01 (2.82–3.21)	1.97 (1.79–2.17)
	2000–2023	5.42 (4.90–5.99)	2.49 (2.45–2.52)	1.60 (1.55–1.64)
Women	2000–2003	5.26 (3.86–7.18)	2.90 (2.77–3.03)	1.63 (1.52–1.74)
	2004–2007	5.50 (4.26–7.11)	2.89 (2.77–3.01)	1.74 (1.63–1.86)
	2008–2011	5.70 (4.57–7.12)	2.89 (2.77–3.01)	1.94 (1.82–2.07)
	2012–2015	11.72 (9.39–14.64)	2.87 (2.75–3.00)	2.18 (2.03–2.33)
	2016–2019	14.42 (10.88–19.11)	2.87 (2.72–3.03)	2.18 (2.02–2.35)
	2020–2023	7.92 (5.55–11.31)	2.85 (2.61–3.11)	2.35 (2.11–2.61)
	2000–2023	6.85 (6.15–7.62)	2.89 (2.83–2.94)	1.81 (1.76–1.87)

*Note:* Cells show age‐standardized rate ratios (RRs) with 95% confidence intervals comparing individuals with bipolar disorder with the general population in Finland within each age group and sex.

In the 25–64‐year group, rate ratios were 2.49 (95% CI 2.45–2.52) among men and 2.89 (95% CI 2.83–2.94) among women. Among those aged 65–74 years, estimates were 1.60 (95% CI 1.55–1.64) and 1.81 (95% CI 1.76–1.87), respectively.

Results from sensitivity analyses using 5‐year age bands within ages 20–64 years were consistent with the main findings, showing the highest relative excess mortality in the youngest adult age groups and a progressive decline with increasing age (Table S3).

## 4 Discussion

The present nationwide register‐based study found that individuals with bipolar disorder in Finland experienced substantially elevated all‐cause mortality compared to the general population during 2000–2023. Excess mortality was observed in both men and women and across all age groups. Although mortality declined over time in both the general population and among individuals with bipolar disorder, the decline was steeper in the bipolar disorder group, resulting in a significant reduction in relative excess mortality over the study period.

The magnitude of excess mortality observed in this study is consistent with previous literature. A meta‐analysis of 15 studies estimated standardized mortality ratios of 2.17 (95% CI 2.01–2.34) for men and 2.11 (95% CI 1.93–2.31) for women with bipolar disorder [10]. In the present study, the age‐standardized mortality rate ratios for 2000–2023 were 2.85 (95% CI 2.77–2.92) among men and 3.25 (95% CI 3.14–3.36) among women. Although these measures are not directly equivalent, the overall magnitude of excess mortality is comparable and somewhat higher in the present study. This consistency underscores the persistent concern of excess mortality among individuals with bipolar disorder. However, our findings regarding time trends differ from several earlier reports.

Mortality declined over time among individuals with bipolar disorder and in the general population. However, the decline was steeper among individuals with bipolar disorder, resulting in a reduction in relative excess mortality during 2000–2023. This finding contrasts with several earlier studies reporting a widening mortality gap, as mortality declined more slowly among individuals with bipolar disorder than in the general population [19, 20, 23]. For instance, a study employing Swedish register‐based data from 1987 to 2010 found that all‐cause mortality among hospitalized individuals with bipolar disorder declined by 9%, while the decline for the general population was 30% [20].

The reason for this inconsistency in the observed trends remains unclear. However, because the present study included both hospitalized and non‐hospitalized individuals with bipolar disorder, who may have milder symptoms and greater engagement with healthcare, our findings may better reflect mortality patterns in the broader bipolar disorder population. Previous research suggests that studies relying exclusively on inpatient data may overestimate mortality among individuals with bipolar disorder [26, 27]. Nevertheless, inclusion of both inpatients and outpatients does not fully account for the differing mortality trends observed across studies, as investigations with similar patient populations have also reported varying results [19, 23].

In addition, substantial changes in the diagnostic criteria for bipolar disorder over recent decades have aimed to enhance diagnostic accuracy and reflect the complexity of the disorder [2, 28]. Increased awareness and recognition of bipolar disorder, along with reduced stigma, may also have contributed to the identification of individuals with less severe forms of the disorder. In the present study, the proportion of the population diagnosed with bipolar disorder increased over time, which may partly reflect broader case identification. It is therefore possible that inclusion of individuals with milder illness has reduced the overall severity of the diagnosed population and, consequently, attenuated relative excess mortality at the population level.

Lastly, the observed reduction in relative excess mortality may also reflect improvements in healthcare and mental health services. Finland has a long‐standing tradition of egalitarian health and welfare policies, with continued investments in mental health care during recent decades [25]. Such developments may have contributed to improved access to care and management of both psychiatric and somatic comorbidities among individuals with bipolar disorder.

A recent Finnish register‐based study identified external causes of death as a major contributor to premature mortality among persons with bipolar disorder aged 15 to 64 years, with alcohol‐related factors playing a prominent role [18]. Although the present study did not examine cause‐specific mortality and cannot determine how external or natural causes contributed to the observed trends, these findings underscore the importance of understanding how different causes of death contribute to excess mortality in bipolar disorder. To better identify targets for prevention, future research should examine how external and natural causes contribute to excess mortality and how the distribution of causes of death has evolved over time.

In the present study, women with bipolar disorder exhibited higher relative excess mortality than men with bipolar disorder. Previous research has been inconsistent regarding whether sex modifies excess mortality in bipolar disorder [8, 24, 29, 30]. The reason for the modifying effect of sex is unclear. However, one possible explanation is that the relative impact of bipolar disorder appears greater in women because baseline mortality rates are substantially higher among men in the general population, resulting in a smaller proportional increase associated with bipolar disorder.

The present study also identified an age‐related pattern in relative excess mortality, consistent with previous research [8, 29, 30, 31, 32]. Young individuals with bipolar disorder exhibited higher relative excess mortality than their older counterparts throughout the study period. Notably, evidence consistently indicates a decrease in relative excess mortality in persons with bipolar disorder with advancing age; as individuals with bipolar disorder age, their mortality increasingly resembles that of the general population, resulting in a smaller relative mortality gap in older age groups [10, 23]. This age difference was also observed in sensitivity analyses using narrower age bands. Such age‐related differences have been attributed to survival effects, as individuals who survive to older age may exhibit better overall health and more established relationships with healthcare providers, leading to improved management of bipolar disorder and possible medical comorbidities [31]. Although this study did not differentiate between early‐ and late‐onset bipolar disorder, the observed reduction in relative excess mortality with age could, in part, be due to symptomatic differences between these two onset patterns.

To our knowledge, previous research has not examined time trends in the mortality gap between individuals with bipolar disorder and the general population, stratified simultaneously by both age group and sex. Our study found that time trends in mortality rates differed across these groups. In the general population, mortality declined more markedly among men than among women across age groups. Among individuals with bipolar disorder, patterns were more heterogeneous: In the youngest age group, mortality declined more among men than women, whereas in the 25–64‐year and 65–74‐year groups, larger declines were observed among women. Reductions in relative excess mortality were most evident in adults aged 25–64 years in both sexes and among women aged 65–74 years, while changes in the youngest age group were less consistent.

The observed heterogeneity in temporal trends may reflect differences in underlying age‐ and sex‐specific mortality levels, healthcare utilization, and the distribution of natural and external causes of death across groups. Improvements were most evident in adults aged 25–64 years, a group that may have more sustained engagement with healthcare services and greater opportunities for prevention and management of somatic comorbidities. In contrast, mortality in the youngest age group is relatively rare and often driven by external causes [18, 33], resulting in less stable estimates and potentially smaller reductions in relative excess mortality over time, as preventable somatic causes of death are less common at younger ages. Among older men, high background mortality and greater overall disease burden may constrain further reductions in relative excess mortality over time. However, as the present study did not examine cause‐specific mortality, the mechanisms underlying these patterns cannot be determined.

Several mechanisms may underlie the association between bipolar disorder and excess mortality. Behavioral and psychological risk factors associated with substantial medical comorbidity and mortality from both external and natural causes are prevalent among individuals with bipolar disorder [32]. Furthermore, physiological factors, such as a shared genetic predisposition, effects on the immune system, and low‐grade inflammation, may contribute to the increased mortality observed in individuals with bipolar disorder [34, 35, 36]. While pharmacological treatments for bipolar disorder have been found to improve quality of life and reduce all‐cause mortality and suicide risk [37], they may also induce metabolic changes that increase the risk of medical comorbidities, potentially offsetting some of these benefits [38]. This underscores the importance of careful treatment selection and ongoing monitoring. Moreover, research indicates that recognizing and treating somatic illnesses and risk factors for external mortality in psychiatric patients may be compromised by patients and medical staff, potentially exacerbating mortality disparities [39]. Addressing these factors is essential to reducing excess mortality associated with bipolar disorder. Future efforts should prioritize both prevention of external causes of death and reduction of mortality from natural causes, which remain substantial in this population.

### 4.1 Strengths and Limitations

The strengths of this study include nationwide population coverage and the use of high‐quality register data. The Hospital Discharge Register has been found to have good accuracy and coverage [40], and Finnish mortality data are considered reliable and virtually complete [25, 41]. Nevertheless, regional variation in diagnostic practices for bipolar disorder within Finland should be acknowledged, which may introduce some heterogeneity in case identification [42].

Several limitations should be considered when interpreting these findings. First, the study relied on specialized in‐ and outpatient register data and did not include individuals treated exclusively in primary care or the private sector, nor those never in contact with healthcare services. Consequently, some individuals with milder or undiagnosed bipolar disorder may have been classified within the general population, potentially biasing effect estimates. Second, the analyses did not include information on socioeconomic status, lifestyle factors, or medical or psychiatric comorbidities, and residual confounding by unmeasured factors cannot be excluded. Third, the data were not differentiated according to cause of death or bipolar spectrum subtype, precluding assessment of cause‐specific mortality and potential differences across bipolar subtypes. Fourth, although sensitivity analyses using narrower adult age bands yielded consistent patterns, the main analyses relied on predefined age categories, which may mask some heterogeneity. Finally, the findings may not be generalizable to other healthcare systems or populations.

## 5 Conclusion

This study showed that individuals with bipolar disorder in Finland continue to face a substantial burden of excess mortality compared with the general population. Although mortality declined over time and relative excess mortality decreased during 2000–2023, improvements were not uniform across age and sex groups. While advancements in healthcare and mental health services may have contributed to these trends, multiple interacting factors are likely involved. Despite these improvements, the persistent excess mortality associated with bipolar disorder underscores the ongoing need for enhanced preventive care, timely management of somatic comorbidities, and targeted efforts to reduce deaths from external causes in this population. Future healthcare development should prioritize these objectives to reduce the excess mortality burden among individuals with bipolar disorder.

## Funding

This work was supported by Signe ja Ane Gyllenbergin Säätiö (5688, 5110), European Research Council (101040247), and the Academy of Finland (354237, 339390).

## Conflicts of Interest

The authors declare no conflicts of interest.

## Supporting information


**Table S1:** Age‐standardized all‐cause mortality rates by period, sex, and population group, 2000–2023.
**Table S2:** Age‐standardized all‐cause mortality rates by age group, period, sex, and population group, 2000–2023.
**Table S3:** Sensitivity analysis of all‐cause mortality rates and rate ratios using 5‐year age bands among individuals aged 20–64 years, 2000–2023.

## Data Availability

The data that support the findings of this study are available from Statistics of Finland and Finnish Institute for Health and Welfare. Restrictions apply to the availability of these data, which were used under license for this study.

## References

[1] K. R. Merikangas , H. S. Akiskal , J. Angst , et al., “Lifetime and 12‐Month Prevalence of Bipolar Spectrum Disorder in the National Comorbidity Survey Replication,” Archives of General Psychiatry 64 (2007): 543–552. 17485606 10.1001/archpsyc.64.5.543 PMC1931566

[2] American Psychiatric Association , Diagnostic and Statistical Manual of Mental Disorders, 2013, 5th ed., (American Psychiatric Association, 2013).

[3] C. Hakulinen , M. Elovainio , M. Arffman , et al., “Mental Disorders and Long‐Term Labour Market Outcomes: Nationwide Cohort Study of 2 055 720 Individuals,” Acta Psychiatrica Scandinavica 140 (2019): 371–381. 31254386 10.1111/acps.13067

[4] C. Hakulinen , M. Elovainio , M. Arffman , et al., “Employment Status and Personal Income Before and After Onset of a Severe Mental Disorder: A Case‐Control Study,” Psychiatric Services 71 (2020): 250–255. 31722646 10.1176/appi.ps.201900239

[5] C. Hakulinen , K. L. Musliner , and E. Agerbo , “Bipolar Disorder and Depression in Early Adulthood and Long‐Term Employment, Income, and Educational Attainment: A Nationwide Cohort Study of 2390,127 Individuals,” Depression and Anxiety 36 (2019): 1080–1088. 31508865 10.1002/da.22956

[6] L. J. Robinson , J. M. Thompson , P. Gallagher , et al., “A Meta‐Analysis of Cognitive Deficits in Euthymic Patients With Bipolar Disorder,” Journal of Affective Disorders 93 (2006): 105–115. 16677713 10.1016/j.jad.2006.02.016

[7] C. Samamé , D. J. Martino , and S. A. Strejilevich , “Social Cognition in Euthymic Bipolar Disorder: Systematic Review and Meta‐Analytic Approach,” Acta Psychiatrica Scandinavica 125 (2012): 266–280. 22211280 10.1111/j.1600-0447.2011.01808.x

[8] C. Crump , K. Sundquist , M. A. Winkleby , and J. Sundquist , “Comorbidities and Mortality in Bipolar Disorder: A Swedish National Cohort Study,” JAMA Psychiatry 70 (2013): 931–939. 23863861 10.1001/jamapsychiatry.2013.1394

[9] J. K. N. Chan , C. C. H. Y. Tong , C. S. M. Wong , E. Y. H. Chen , and W. C. Chang , “Life Expectancy and Years of Potential Life Lost in Bipolar Disorder: Systematic Review and Meta‐Analysis,” British Journal of Psychiatry 221 (2022): 567–576. 10.1192/bjp.2022.19 35184778

[10] J. F. Hayes , J. Miles , K. Walters , M. King , and D. P. J. Osborn , “A Systematic Review and Meta‐Analysis of Premature Mortality in Bipolar Affective Disorder,” Acta Psychiatrica Scandinavica 131 (2015): 417–425. 25735195 10.1111/acps.12408 PMC4939858

[11] J. Oeppen and J. W. Vaupel , “Broken Limits to Life Expectancy,” Science 296 (2002): 1029–1031. 12004104 10.1126/science.1069675

[12] World Bank , Life expectancy at birth (total), 2026 accessed at, February 23, 2026, https://data.worldbank.org/indicator/SP.DYN.LE00.IN.

[13] C. K. Chang , R. D. Hayes , G. Perera , et al., “Life Expectancy at Birth for People With Serious Mental Illness and Other Major Disorders From a Secondary Mental Health Care Case Register in London,” PLoS One 6 (2011): e19590. 21611123 10.1371/journal.pone.0019590 PMC3097201

[14] T. M. Laursen , K. Wahlbeck , J. Hällgren , et al., “Life Expectancy and Death by Diseases of the Circulatory System in Patients With Bipolar Disorder or Schizophrenia in the Nordic Countries,” PLoS One 8 (2013): e67133. 23826212 10.1371/journal.pone.0067133 PMC3691116

[15] T. M. Laursen , “Life Expectancy Among Persons With Schizophrenia or Bipolar Affective Disorder,” Schizophrenia Research 131 (2011): 101–104. 21741216 10.1016/j.schres.2011.06.008

[16] M. Nordentoft , K. Wahlbeck , J. Hällgren , et al., “Excess Mortality, Causes of Death and Life Expectancy in 270,770 Patients With Recent Onset of Mental Disorders in Denmark, Finland and Sweden,” PLoS One 8 (2013): e55176. 23372832 10.1371/journal.pone.0055176 PMC3555866

[17] B. Roshanaei‐Moghaddam and W. Katon , “Premature Mortality From General Medical Illnesses Among Persons With Bipolar Disorder: A Review,” Psychiatric Services 60 (2009): 147–156. 19176408 10.1176/ps.2009.60.2.147

[18] T. Paljärvi , K. Herttua , H. Taipale , et al., “Cause‐Specific Excess Mortality After First Diagnosis of Bipolar Disorder: Population‐Based Cohort Study,” BMJ Ment Health 26 (2023): e300700. 10.1136/bmjment-2023-300700 PMC10391789 37463759

[19] J. F. Hayes , L. Marston , K. Walters , M. B. King , and D. P. J. Osborn , “Mortality Gap for People With Bipolar Disorder and Schizophrenia: UK‐Based Cohort Study 2000‐2014,” British Journal of Psychiatry 211 (2017): 175–181. 10.1192/bjp.bp.117.202606 PMC5579328 28684403

[20] U. Ösby , J. Westman , J. Hällgren , and M. Gissler , “Mortality Trends in Cardiovascular Causes in Schizophrenia, Bipolar and Unipolar Mood Disorder in Sweden 1987‐2010,” European Journal of Public Health 26 (2016): 867–871. 26748100 10.1093/eurpub/ckv245 PMC5054269

[21] T. M. Laursen , T. Munk‐Olsen , and C. Gasse , “Chronic Somatic Comorbidity and Excess Mortality due to Natural Causes in Persons With Schizophrenia or Bipolar Affective Disorder,” PLoS One 6 (2011): e24597. 21935426 10.1371/journal.pone.0024597 PMC3173467

[22] L. H. Lomholt , D. V. Andersen , C. Sejrsgaard‐Jacobsen , et al., “Mortality Rate Trends in Patients Diagnosed With Schizophrenia or Bipolar Disorder: A Nationwide Study With 20 Years of Follow‐Up,” Int J Bipolar Disord 7 (2019): 1. 30820700 10.1186/s40345-018-0140-x PMC6395457

[23] P. Staudt Hansen , M. Frahm Laursen , S. Grøntved , S. Puggard Vogt Straszek , R. W. Licht , and R. E. Nielsen , “Increasing Mortality Gap for Patients Diagnosed With Bipolar Disorder—A Nationwide Study With 20 Years of Follow‐Up,” Bipolar Disorders 21 (2019): 270–275. 30051555 10.1111/bdi.12684

[24] U. Ösby , L. Brandt , N. Correia , A. Ekbom , and P. Sparén , “Excess Mortality in Bipolar and Unipolar Disorder in Sweden,” Bipolar Disorders 3 (2001): 22–28. 10.1001/archpsyc.58.9.844 11545667

[25] K. Wahlbeck , J. Westman , M. Nordentoft , M. Gissler , and T. M. Laursen , “Outcomes of Nordic Mental Health Systems: Life Expectancy of Patients With Mental Disorders,” British Journal of Psychiatry 199 (2011): 453–458. 10.1192/bjp.bp.110.085100 21593516

[26] C. Crump , J. P. A. Ioannidis , K. Sundquist , M. A. Winkleby , and J. Sundquist , “Mortality in Persons With Mental Disorders Is Substantially Overestimated Using Inpatient Psychiatric Diagnoses,” Journal of Psychiatric Research 47 (2013): 1298–1303. 23806577 10.1016/j.jpsychires.2013.05.034 PMC3746500

[27] K. Suokas , C. Hakulinen , R. Sund , O. Kampman , and S. Pirkola , “Mortality in Persons With Recent Primary or Secondary Care Contacts for Mental Disorders in Finland,” World Psychiatry 21 (2022): 470–471. 36073698 10.1002/wps.21027 PMC9453896

[28] International Classification of Diseases Eleventh Revision (ICD‐11), (World Health Organization, 2022).

[29] U. Hoang , R. Stewart , and M. J. Goldacre , “Mortality After Hospital Discharge for People With Schizophrenia or Bipolar Disorder: Retrospective Study of Linked English Hospital Episode Statistics, 1999‐2006,” BMJ 343 (2011): d5422. 21914766 10.1136/bmj.d5422 PMC3172324

[30] T. M. Laursen , T. Munk‐Olsen , M. Nordentoft , and P. B. Mortensen , “Increased Mortality Among Patients Admitted With Major Psychiatric Disorders: A Register‐Based Study Comparing Mortality in Unipolar Depressive Disorder, Bipolar Affective Disorder, Schizoaffective Disorder, and Schizophrenia,” Journal of Clinical Psychiatry 68 (2007): 899–907. 17592915 10.4088/jcp.v68n0612

[31] C. K. Chang , R. D. Hayes , M. Broadbent , et al., “All‐Cause Mortality Among People With Serious Mental Illness, Substance Use Disorders, and Depressive Disorders in Southeast London: A Cohort Study,” BMC Psychiatry 10 (2010): 77. 20920287 10.1186/1471-244X-10-77 PMC2958993

[32] L. V. Kessing , E. Vradi , M. I. RS , and P. K. Andersen , “Causes of Decreased Life Expectancy Over the Life Span in Bipolar Disorder,” Journal of Affective Disorders 180 (2015): 142–147. 25909752 10.1016/j.jad.2015.03.027

[33] A. Partanen , J. Moring , V. Bergman , et al., Miten tästä eteenpäin? Työpaperi 20/2015, 2015, (Terveyden ja hyvinvoinnin laitos, 2015).

[34] R. A. Marrie , R. Walld , J. M. Bolton , et al., “Increased Incidence of Psychiatric Disorders in Immune‐Mediated Inflammatory Disease,” Journal of Psychosomatic Research 101 (2017): 17–23. 28867419 10.1016/j.jpsychores.2017.07.015

[35] J. D. Rosenblat and R. S. McIntyre , “Bipolar disorder and inflammation,” Psychiatric Clinics of North America 39 (2016): 125–137. 26876323 10.1016/j.psc.2015.09.006

[36] L. Y. Wang , J. H. Chiang , S. F. Chen , and Y. C. Shen , “Systemic Autoimmune Diseases Are Associated With an Increased Risk of Bipolar Disorder: A Nationwide Population‐Based Cohort Study,” Journal of Affective Disorders 227 (2018): 31–37. 29049933 10.1016/j.jad.2017.10.027

[37] M. Lähteenvuo , A. Tanskanen , H. Taipale , et al., “Psychopharmacological Treatment, Mortality and Suicide in Bipolar Disorder in a Finnish Nationwide Cohort of 18,018 Patients,” Psychiatria Fennica 49 (2018): 10–21.

[38] C. S. Wu , K. Y. Wu , Y. R. Lo , et al., “Psychotropic Use and Risk of Stroke Among Patients With Bipolar Disorders: 10‐Year Nationwide Population‐Based Study,” Journal of Affective Disorders 226 (2018): 77–84. 28964996 10.1016/j.jad.2017.09.020

[39] T. M. Laursen and M. Nordentoft , “Heart Disease Treatment and Mortality in Schizophrenia and Bipolar Disorder—Changes in the Danish Population Between 1994 and 2006,” Journal of Psychiatric Research 45 (2011): 29–35. 20546788 10.1016/j.jpsychires.2010.04.027

[40] J. Haukka , “Finnish Health and Social Welfare Registers in Epidemiological Research,” Norsk Epidemiologi 14 (2004): 113–120.

[41] R. A. Lahti and A. Penttilä , “The Validity of Death Certificates: Routine Validation of Death Certification and Its Effects on Mortality Statistics,” Forensic Science International 115 (2001): 15–32. 11056267 10.1016/s0379-0738(00)00300-5

[42] K. Suokas , O. Kurkela , J. Nevalainen , et al., “Geographical Variation in Treated Psychotic and Other Mental Disorders in Finland by Region and Urbanicity,” Social Psychiatry and Psychiatric Epidemiology 59 (2024): 37–49. 37308692 10.1007/s00127-023-02516-x PMC10799825

