# Supplementary material for: Time Trends in Excess Mortality Among Individuals With Bipolar Disorder in Finland, 2000–2023: A Nationwide Register Study
Source: Bipolar Disord. 2026 Jul 5;28(5):e70147. doi: 10.1111/bdi.70147 (PMC13334196; doi:10.1111/bdi.70147)
Supplement: Supplementary file 1 — Table S1: Age‐standardized all‐cause mortality rates by period, sex, and population group, 2000–2023. Table S2: Age‐standardized all‐cause mortality rates by age group, period, sex, and population group, 2000–2023. Table S3: Sensitivity analysis of all‐cause mortality rates and rate ratios using 5‐year age bands among individuals aged 20–64 years, 2000–2023. [file BDI-28-0-s001.docx]

**Supplementary Material for:**

Time Trends in Excess Mortality Among Individuals with Bipolar Disorder in Finland, 2000–2023: A Nationwide Register Study 

**Supplementary Results**

J. Rimmington, S. Lumme, K. Suokas, M. Gutvilig, M. Elovainio & C. Hakulinen

[Supplementary Table S1. Age-standardized all-cause mortality rates by period, sex, and population group, 2000–2023.](#_Toc233276848)

[Supplementary Table S2. Age-standardized all-cause mortality rates by age group, period, sex, and population group, 2000–2023.](#_Toc233276849)

[Supplementary Table S3. Sensitivity analysis of all-cause mortality rates and rate ratios using 5-year age bands among individuals aged 20–64 years, 2000–2023.](#_Toc233276850)

Supplementary Table S1. Age-standardized all-cause mortality rates by period, sex, and population group, 2000–2023.

|  | **Bipolar disorder population** | | **General population** | |
| --- | --- | --- | --- | --- |
| **Period** | **Men** | **Women** | **Men** | **Women** |
| 2000–2003 | 2,201 (2,005–2,416) | 1,336 (1,199–1,488) | 747 (741–754) | 328 (324–332) |
| 2004–2007 | 2,060 (1,910–2,221) | 1,014 (919–1,120) | 687 (681–693) | 303 (299–307) |
| 2008–2011 | 1,843 (1,725–1,969) | 929 (854–1,010) | 626 (621–632) | 282 (278–285) |
| 2012–2015 | 1,429 (1,341–1,524) | 753 (695–816) | 553 (548–558) | 263 (259–266) |
| 2016–2019 | 1,395 (1,315–1,480) | 693 (643–746) | 507 (503–512) | 250 (247–253) |
| 2020–2023 | 1,361 (1,287–1,439) | 709 (662–759) | 499 (495–504) | 252 (248–255) |
| 2000–2023 | 13,742 (13,375–14,118) | 7,258 (7,017–7,507) | 4829 (4811–4847) | 2,236 (2,224–2,248) |

Age-standardized all-cause mortality rates are per 100,000 person-years with 95% confidence intervals, stratified by period, sex, and population group.

Supplementary Table S2. Age-standardized all-cause mortality rates by age group, period, sex, and population group, 2000–2023.

|  |  | **Bipolar disorder population** | | **General population** | |
| --- | --- | --- | --- | --- | --- |
| **Age group** | **Period** | **Men** | **Women** | **Men** | **Women** |
| 10–24 | 2000–2003 | 710 (382–1,320) | 328 (164–656) | 69 (65–72) | 24 (22–26) |
|  | 2004–2007 | 528 (324–863) | 187 (94–375) | 63 (60–67) | 25 (23–27) |
|  | 2008–2011 | 238 (141–401) | 243 (160–369) | 59 (55–62) | 21 (19–23) |
|  | 2012–2015 | 270 (168–434) | 84 (47–152) | 45 (42–48) | 19 (18–22) |
|  | 2016–2019 | 349 (230–530) | 135 (87–210) | 45 (42–48) | 22 (20–24) |
|  | 2020–2023 | 277 (175–440) | 155 (106–228) | 50 (47–53) | 22 (20–24) |
|  | 2000–2023 | 3,190 (2,614–3,892) | 1,473 (1,205–1,801) | 441 (431–452) | 178 (171–185) |
| 25–64 | 2000–2003 | 1,720 (1,539–1,922) | 1,063 (928–1,219) | 526 (520–532) | 225 (221–229) |
|  | 2004–2007 | 1,848 (1,697–2,011) | 852 (757–958) | 513 (508–519) | 219 (215–223) |
|  | 2008–2011 | 1,551 (1,437–1,675) | 792 (718–873) | 465 (459–470) | 202 (199–206) |
|  | 2012–2015 | 1,242 (1,152–1,340) | 608 (551–671) | 398 (393–403) | 180 (177–184) |
|  | 2016–2019 | 1,173 (1,090–1,262) | 505 (457–559) | 352 (347–357) | 168 (164–171) |
|  | 2020–2023 | 1,080 (1,004–1,162) | 527 (480–578) | 338 (333–343) | 163 (160–166) |
|  | 2000–2023 | 11,477 (11,109–11,858) | 5,817 (5,574–6,071) | 3,456 (3,438–3,473) | 1,542 (1,530–1,553) |
| 65–74 | 2000–2003 | 7,046 (5,905–8,408) | 4,342 (3,614–5,217) | 2,966 (2,925–3,007) | 1,344 (1,320–1,369) |
|  | 2004–2007 | 5,611 (4,712–6,682) | 3,175 (2,634–3,827) | 2,576 (2,539–2,613) | 1,176 (1,153–1,199) |
|  | 2008–2011 | 5,916 (5,162–6,779) | 2,729 (2,291–3,252) | 2,359 (2,326–2,393) | 1,106 (1,084–1,127) |
|  | 2012–2015 | 4,248 (3,746–4,817) | 2,571 (2,230–2,964) | 2,160 (2,131–2,190) | 1,072 (1,053–1,092) |
|  | 2016–2019 | 4,207 (3,790–4,669) | 2,540 (2,263–2,852) | 2,040 (2,014–2,067) | 1,035 (1,017–1,053) |
|  | 2020–2023 | 4,531 (4,147–4,950) | 2,520 (2,269–2,799) | 2,043 (2,017–2,069) | 1,070 (1,053–1,088) |
|  | 2000–2023 | 42,255 (40,198–44,418) | 23,897 (22,596–25,303) | 18,864 (18,759–18,969) | 9,074 (9,007–9,141) |

Age-standardized all-cause mortality rates are per 100,000 person-years with 95% confidence intervals, stratified by age group, period, sex, and population group.

Supplementary Table S3. Sensitivity analysis of all-cause mortality rates and rate ratios using 5-year age bands among individuals aged 20–64 years, 2000–2023.

| **Sex** | **Age group** | **Bipolar disorder population** | **General population** | **Rate ratio** |
| --- | --- | --- | --- | --- |
| Men | 20–24 | 647 (523–802) | 90 (87–93) | 7.17 (5.77–8.90) |
|  | 25–29 | 704 (601–825) | 92 (89–95) | 7.65 (6.51–8.99) |
|  | 30–34 | 657 (568–760) | 107 (104–110) | 6.12 (5.28–7.10) |
|  | 35–39 | 819 (725–925) | 145 (142–149) | 5.64 (4.98–6.39) |
|  | 40–44 | 970 (871–1,080) | 220 (215–224) | 4.41 (3.96–4.92) |
|  | 45–49 | 1,212 (1,104–1,330) | 351 (346–357) | 3.45 (3.14–3.79) |
|  | 50–54 | 1,653 (1,530–1,785) | 543 (537–550) | 3.04 (2.81–3.29) |
|  | 55–59 | 2,130 (1,987–2,283) | 828 (819–836) | 2.57 (2.40–2.76) |
|  | 60–64 | 2,626 (2,451–2,814) | 1,244 (1,233–1,255) | 2.11 (1.97–2.26) |
| Women | 20–24 | 294 (236–366) | 30 (29–32) | 9.68 (7.72–12.14) |
|  | 25–29 | 202 (162–252) | 31 (30–33) | 6.44 (5.13–8.08) |
|  | 30–34 | 235 (193–286) | 40 (38–42) | 5.90 (4.82–7.23) |
|  | 35–39 | 320 (270–379) | 62 (59–64) | 5.20 (4.37–6.18) |
|  | 40–44 | 439 (379–509) | 98 (95–101) | 4.47 (3.85–5.20) |
|  | 45–49 | 573 (504–651) | 158 (155–162) | 3.62 (3.18–4.12) |
|  | 50–54 | 825 (744–914) | 250 (246–255) | 3.30 (2.97–3.66) |
|  | 55–59 | 1,032 (941–1,133) | 375 (369–380) | 2.76 (2.51–3.03) |
|  | 60–64 | 1,566 (1,444–1,699) | 564 (556–571) | 2.78 (2.56–3.02) |

Age-standardized all-cause mortality rates are presented per 100,000 person-years with 95% confidence intervals. Rate ratios compare the bipolar disorder population with the general population within each age band and sex.
